# 3-Anilinothio­carbonyl-4-hydroxy­chromen-2-one

**DOI:** 10.1107/S1600536809011246

**Published:** 2009-03-31

**Authors:** Rajni Kant, Lovely Sarmal, Sabeta Kohli, Kamni, Mehtab Parveen

**Affiliations:** a Department of Physics, University of Jammu, Jammu Tawi 180 006, India; b School of Applied Physics and Mathematics, Shri Mata Vaishno Devi University, Jammu 182 121, India; c Department of Chemistry, Aligarh Muslim University, Aligarh, Uttar Pradesh 202 002, India

## Abstract

The geometrical parameters of the title compound, C_16_H_11_NO_3_S, are in the usual ranges. The two aromatic residues are not coplanar and are twisted by a dihedral angle of 66.63 (6)°. The crystal structure is stabilized by N—H⋯O and  O—H⋯S inter­actions.

## Related literature

For literature on coumarins, see: Campbell (1959 ▶); Murray *et al.* (1982 ▶); Wolska *et al.* (1990 ▶); Harvey (1999 ▶); Matern *et al.* (1999 ▶); Yang *et al.* (1992 ▶); Tsai *et al.* (2000 ▶).
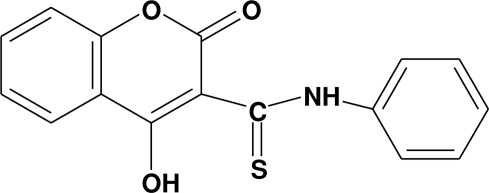


## Experimental

### 

#### Crystal data

C_16_H_11_NO_3_S
*M* *_r_* = 297.33
Monoclinic, 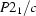

*a* = 14.8059 (9) Å
*b* = 5.5245 (4) Å
*c* = 17.4438 (12) Å
β = 109.091 (7)°
*V* = 1348.34 (16) Å^3^
*Z* = 4
Mo *K*α radiation
μ = 0.25 mm^−1^
*T* = 293 K
0.30 × 0.24 × 0.18 mm

#### Data collection

Oxford Diffraction Xcalibur diffractometer
Absorption correction: none
11450 measured reflections
4408 independent reflections
2320 reflections with *I* > 2σ(*I*)
*R*_int_ = 0.036

#### Refinement

*R*[*F* ^2^ > 2σ(*F* ^2^)] = 0.049
*wR*(*F* ^2^) = 0.134
*S* = 1.01
4408 reflections
235 parameters
All H-atom parameters refined
Δρ_max_ = 0.24 e Å^−3^
Δρ_min_ = −0.23 e Å^−3^

### 

Data collection: *CrysAlisPro* (Oxford Diffraction, 2007 ▶); cell refinement: *CrysAlisPro*; data reduction: *CrysAlis RED* (Oxford Diffraction, 2007 ▶); program(s) used to solve structure: *SHELXS86* (Sheldrick, 2008 ▶); program(s) used to refine structure: *SHELXL97* (Sheldrick, 2008 ▶); molecular graphics: *ORTEP-3 for Windows* (Farrugia, 1997 ▶); software used to prepare material for publication: *WinGX* (Farrugia, 1999 ▶).

## Supplementary Material

Crystal structure: contains datablocks global, I. DOI: 10.1107/S1600536809011246/jh2075sup1.cif

Structure factors: contains datablocks I. DOI: 10.1107/S1600536809011246/jh2075Isup2.hkl

Additional supplementary materials:  crystallographic information; 3D view; checkCIF report

## Figures and Tables

**Table 1:** Hydrogen-bond geometry (Å, °)

*D*—H⋯*A*	*D*—H	H⋯*A*	*D*⋯*A*	*D*—H⋯*A*
N1—H1*A*⋯O2	0.96 (2)	1.77 (2)	2.5923 (19)	141 (2)
O3—H3*A*⋯S1	1.05 (2)	1.81 (3)	2.8163 (15)	159 (2)

**Fig. 1.**
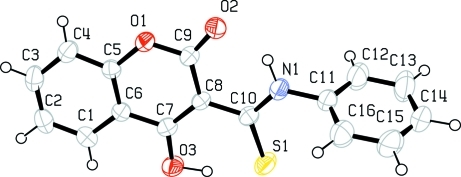
View of (I) (50% probability displacement ellipsoids)

**Fig. 2.**
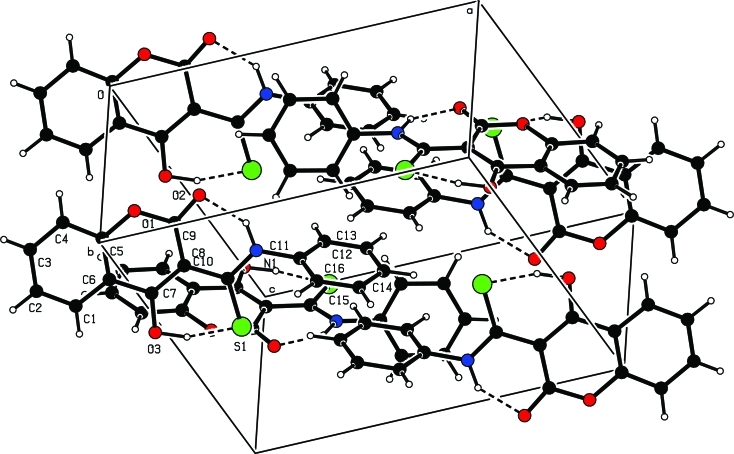
Depiction of X—H···A interactions in the title compound(I)

**Fig. 3.**
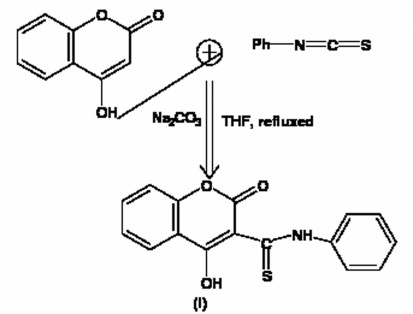
The synthesis of the title compound.

## supplementary crystallographic information

### Comment

Coumarins belong to a group of compounds known as benzopyrones, all of which consist of a benzene ring joined to a pyrone moiety.

Coumarins are found to have a wide spectrum of biological activity, *e.g.* antithronbotic effect, vascodilating effect on vessel, tonic influence on capillary blood vessels, reduction of blood pressure, antispastic and photosensitizing effect (Wolska *et al.*, 1990).

Interestingly, coumarins exhibit inhibitory effect on DNA gyrase, which may be linked to anti-HIV (human immuno deficiency virus) activity (Matern *et al.*, 1999).

Coumarins are also found to exhibit anti-malarial activity (Yang *et al.*, 1992).

Recently Collinin, isolated from Zathoxylum Schinifolium, has been found to exhibit anti-HBV (hepatitis B virus) activity (Tsai *et al.*, 2000).

Owing to the general importance of these coumarin analogues we report herein the synthesis and crystal structure of a new coumarin 3-anilinothiocarbonyl-4-hydroxychromen-2-one, (I).

The geometrical parameters (*i.e.* bond distances and angles) of (I) are in the usual ranges. The two aromatic residues are not coplanar and are twisted by a dihedral angle of 66.63 (6)°. The crystal structure is stabilized by *X*—H···A interactions.

### Experimental

Scheme1: The mixture of 4-hydroxy coumarin, phenylisothiocyanate was taken in THF.The base Na_2_CO_3_ was also added to it. The reaction mixture was refluxed on water bath for three hours. Progress of the reaction was monitored by TLC. After completion the reaction mixture was poured into water and worked up with ether and then in ethyl acetate. The ether layer showed the presence of three compounds from which the title compound (I) was separated by column chromatography followed by crystallization from chloroform-methanol as white crystalline solid. Melting point: 421–423 K.

### Refinement

All H atoms were located from difference Fourier map and refined isotopically with distance restraints 0.86–1.05 Å.

### Crystal data

**Table:**

C_16_H_11_NO_3_S	*F*(000) = 616
*M**_r_* = 297.33	*D*_x_ = 1.465 Mg m^−^^3^
Monoclinic, *P*2_1_/*c*	Mo *K*α radiation, λ = 0.71073 Å
*a* = 14.8059 (9) Å	Cell parameters from 2320 reflections
*b* = 5.5245 (4) Å	θ = 2.9–32.3°
*c* = 17.4438 (12) Å	µ = 0.25 mm^−^^1^
β = 109.091 (7)°	*T* = 293 K
*V* = 1348.34 (16) Å^3^	Rectangular, yellow
*Z* = 4	0.30 × 0.24 × 0.18 mm

### Data collection

**Table:**

Oxford Diffraction Xcalibur diffractometer	2320 reflections with *I* > 2σ(*I*)
Radiation source: fine-focus sealed tube	*R*_int_ = 0.036
graphite	θ_max_ = 32.3°, θ_min_ = 2.9°
ω–2θ scans	*h* = −20→21
11450 measured reflections	*k* = −8→5
4408 independent reflections	*l* = −25→26

### Refinement

**Table:**

Refinement on *F*^2^	0 constraints
Least-squares matrix: full	All H-atom parameters refined
*R*[*F*^2^ > 2σ(*F*^2^)] = 0.049	*w* = 1/[σ^2^(*F*_o_^2^) + (0.0645*P*)^2^] where *P* = (*F*_o_^2^ + 2*F*_c_^2^)/3
*wR*(*F*^2^) = 0.134	(Δ/σ)_max_ < 0.001
*S* = 1.01	Δρ_max_ = 0.24 e Å^−^^3^
4408 reflections	Δρ_min_ = −0.23 e Å^−^^3^
235 parameters	Extinction correction: *SHELXL97* (Sheldrick, 2008), Fc^*^=kFc[1+0.001xFc^2^λ^3^/sin(2θ)]^-1/4^
0 restraints	Extinction coefficient: 0.0079 (18)

### Special details

**Table:**

Geometry. All e.s.d.'s (except the e.s.d. in the dihedral angle between two l.s. planes) are estimated using the full covariance matrix. The cell e.s.d.'s are taken into account individually in the estimation of e.s.d.'s in distances, angles and torsion angles; correlations between e.s.d.'s in cell parameters are only used when they are defined by crystal symmetry. An approximate (isotropic) treatment of cell e.s.d.'s is used for estimating e.s.d.'s involving l.s. planes.

### Fractional atomic coordinates and isotropic or equivalent isotropic displacement parameters (Å^2^)

**Table:**

	*x*	*y*	*z*	*U*_iso_*/*U*_eq_	
H3	−0.2431 (14)	−0.089 (4)	−0.0463 (12)	0.058 (6)*	
H1	−0.1035 (12)	0.344 (4)	0.1367 (11)	0.044 (5)*	
H4	−0.1108 (14)	−0.346 (4)	−0.0190 (11)	0.053 (6)*	
H2	−0.2420 (15)	0.255 (4)	0.0323 (13)	0.060 (6)*	
H1A	0.2933 (15)	−0.272 (4)	0.2272 (13)	0.066 (7)*	
H14	0.6547 (16)	−0.057 (4)	0.4609 (13)	0.074 (6)*	
H12	0.4091 (17)	−0.402 (5)	0.3868 (13)	0.080 (8)*	
H15	0.5906 (18)	0.239 (5)	0.3553 (16)	0.095 (9)*	
H16	0.4290 (17)	0.200 (5)	0.2686 (16)	0.084 (8)*	
H13	0.5615 (19)	−0.365 (5)	0.4674 (16)	0.099 (9)*	
H3A	0.127 (2)	0.298 (5)	0.2783 (16)	0.102 (9)*	
S1	0.24977 (3)	0.22219 (9)	0.33460 (3)	0.05005 (18)	
C8	0.14300 (10)	−0.0442 (3)	0.20025 (9)	0.0319 (3)	
O1	0.04858 (8)	−0.2950 (2)	0.08806 (7)	0.0400 (3)	
O2	0.19660 (8)	−0.3959 (2)	0.14831 (7)	0.0497 (3)	
C10	0.23535 (11)	0.0021 (3)	0.26441 (9)	0.0349 (4)	
O3	0.06066 (9)	0.2954 (2)	0.23189 (8)	0.0470 (3)	
C6	−0.02392 (11)	0.0583 (3)	0.12239 (9)	0.0329 (4)	
C7	0.06394 (11)	0.1047 (3)	0.18839 (9)	0.0338 (4)	
N1	0.30848 (10)	−0.1395 (3)	0.26561 (9)	0.0452 (4)	
C5	−0.02859 (11)	−0.1432 (3)	0.07446 (9)	0.0333 (4)	
C1	−0.10418 (12)	0.2061 (3)	0.10584 (12)	0.0423 (4)	
C4	−0.10991 (12)	−0.2023 (4)	0.01022 (11)	0.0420 (4)	
C9	0.13442 (12)	−0.2508 (3)	0.14694 (10)	0.0351 (4)	
C11	0.40436 (12)	−0.1113 (4)	0.32054 (11)	0.0449 (4)	
C2	−0.18535 (13)	0.1526 (4)	0.04245 (12)	0.0494 (5)	
C3	−0.18805 (13)	−0.0511 (4)	−0.00491 (11)	0.0484 (5)	
C16	0.45938 (15)	0.0776 (4)	0.31041 (15)	0.0619 (6)	
C12	0.44115 (17)	−0.2784 (5)	0.37918 (17)	0.0725 (7)	
C15	0.55294 (16)	0.0988 (5)	0.36243 (18)	0.0735 (7)	
C14	0.58922 (15)	−0.0672 (5)	0.42249 (17)	0.0769 (8)	
C13	0.53483 (19)	−0.2533 (5)	0.4308 (2)	0.0947 (11)	

### Atomic displacement parameters (Å^2^)

**Table:**

	*U*^11^	*U*^22^	*U*^33^	*U*^12^	*U*^13^	*U*^23^
S1	0.0422 (3)	0.0555 (3)	0.0476 (3)	−0.0063 (2)	0.00809 (19)	−0.0194 (2)
C8	0.0336 (8)	0.0328 (8)	0.0296 (8)	−0.0035 (7)	0.0105 (6)	−0.0054 (7)
O1	0.0368 (6)	0.0394 (7)	0.0388 (6)	0.0011 (5)	0.0053 (5)	−0.0093 (5)
O2	0.0435 (7)	0.0478 (8)	0.0498 (7)	0.0107 (6)	0.0044 (5)	−0.0145 (6)
C10	0.0351 (8)	0.0355 (9)	0.0351 (8)	−0.0044 (7)	0.0128 (6)	−0.0023 (7)
O3	0.0429 (7)	0.0470 (8)	0.0490 (7)	0.0045 (6)	0.0122 (6)	−0.0170 (6)
C6	0.0341 (8)	0.0331 (9)	0.0323 (8)	−0.0012 (7)	0.0119 (6)	0.0017 (7)
C7	0.0366 (8)	0.0337 (9)	0.0335 (8)	−0.0050 (7)	0.0149 (6)	−0.0015 (7)
N1	0.0335 (7)	0.0466 (9)	0.0490 (9)	−0.0002 (7)	0.0047 (6)	−0.0124 (8)
C5	0.0334 (8)	0.0325 (9)	0.0344 (8)	−0.0012 (7)	0.0113 (6)	0.0034 (7)
C1	0.0434 (10)	0.0391 (10)	0.0451 (10)	0.0058 (8)	0.0154 (8)	0.0049 (9)
C4	0.0413 (9)	0.0444 (11)	0.0373 (9)	−0.0086 (8)	0.0087 (7)	−0.0007 (8)
C9	0.0342 (8)	0.0365 (9)	0.0338 (8)	−0.0028 (7)	0.0099 (6)	−0.0032 (7)
C11	0.0334 (8)	0.0442 (11)	0.0521 (11)	−0.0010 (8)	0.0072 (8)	−0.0101 (9)
C2	0.0370 (9)	0.0545 (13)	0.0525 (11)	0.0086 (9)	0.0088 (8)	0.0123 (10)
C3	0.0347 (9)	0.0594 (13)	0.0439 (10)	−0.0065 (9)	0.0030 (8)	0.0094 (9)
C16	0.0458 (11)	0.0608 (14)	0.0740 (15)	−0.0072 (11)	0.0124 (10)	0.0046 (12)
C12	0.0509 (12)	0.0590 (15)	0.0860 (17)	−0.0148 (11)	−0.0073 (11)	0.0140 (13)
C15	0.0425 (11)	0.0662 (16)	0.110 (2)	−0.0167 (12)	0.0226 (12)	−0.0133 (15)
C14	0.0402 (11)	0.0640 (16)	0.103 (2)	−0.0036 (11)	−0.0091 (12)	−0.0183 (15)
C13	0.0616 (15)	0.0752 (18)	0.105 (2)	−0.0106 (14)	−0.0302 (15)	0.0220 (16)

### Geometric parameters (Å, °)

**Table:**

S1—C10	1.6889 (17)	C1—H1	0.929 (19)
S1—H3A	1.81 (3)	C4—C3	1.381 (3)
C8—C7	1.390 (2)	C4—H4	0.94 (2)
C8—C9	1.452 (2)	C11—C12	1.353 (3)
C8—C10	1.479 (2)	C11—C16	1.370 (3)
O1—C9	1.3696 (19)	C2—C3	1.389 (3)
O1—C5	1.3737 (19)	C2—H2	0.98 (2)
O2—C9	1.215 (2)	C3—H3	0.919 (19)
C10—N1	1.330 (2)	C16—C15	1.391 (3)
O3—C7	1.308 (2)	C16—H16	0.99 (3)
O3—H3A	1.05 (3)	C12—C13	1.392 (3)
C6—C5	1.381 (2)	C12—H12	0.86 (3)
C6—C1	1.392 (2)	C15—C14	1.363 (4)
C6—C7	1.450 (2)	C15—H15	0.98 (3)
N1—C11	1.438 (2)	C14—C13	1.343 (4)
N1—H1A	0.97 (2)	C14—H14	0.98 (2)
C5—C4	1.389 (2)	C13—H13	0.88 (3)
C1—C2	1.373 (3)		
			
C10—S1—H3A	84.7 (8)	O2—C9—O1	114.13 (14)
C7—C8—C9	118.50 (14)	O2—C9—C8	126.81 (15)
C7—C8—C10	122.40 (14)	O1—C9—C8	119.06 (14)
C9—C8—C10	119.10 (14)	C12—C11—C16	120.52 (19)
C9—O1—C5	122.43 (13)	C12—C11—N1	119.68 (18)
N1—C10—C8	117.17 (15)	C16—C11—N1	119.71 (18)
N1—C10—S1	120.18 (12)	C1—C2—C3	120.06 (18)
C8—C10—S1	122.65 (12)	C1—C2—H2	119.6 (13)
C7—O3—H3A	104.8 (15)	C3—C2—H2	120.3 (12)
C5—C6—C1	118.51 (15)	C4—C3—C2	121.14 (17)
C5—C6—C7	118.51 (14)	C4—C3—H3	119.0 (13)
C1—C6—C7	122.98 (16)	C2—C3—H3	119.9 (13)
O3—C7—C8	125.40 (14)	C11—C16—C15	119.2 (2)
O3—C7—C6	114.13 (14)	C11—C16—H16	118.0 (14)
C8—C7—C6	120.45 (15)	C15—C16—H16	122.7 (15)
C10—N1—C11	124.58 (16)	C11—C12—C13	119.4 (2)
C10—N1—H1A	115.4 (13)	C11—C12—H12	123.2 (15)
C11—N1—H1A	120.0 (13)	C13—C12—H12	117.4 (15)
O1—C5—C6	120.90 (14)	C14—C15—C16	120.2 (2)
O1—C5—C4	116.65 (15)	C14—C15—H15	121.9 (15)
C6—C5—C4	122.44 (15)	C16—C15—H15	117.9 (15)
C2—C1—C6	120.24 (19)	C13—C14—C15	119.9 (2)
C2—C1—H1	118.9 (11)	C13—C14—H14	117.6 (13)
C6—C1—H1	120.8 (11)	C15—C14—H14	122.5 (13)
C3—C4—C5	117.60 (18)	C14—C13—C12	120.8 (3)
C3—C4—H4	123.0 (12)	C14—C13—H13	118.1 (18)
C5—C4—H4	119.3 (12)	C12—C13—H13	120.9 (18)

### Hydrogen-bond geometry (Å, °)

**Table:**

*D*—H···*A*	*D*—H	H···*A*	*D*···*A*	*D*—H···*A*
N1—H1A···O2	0.96 (2)	1.77 (2)	2.5923 (19)	141 (2)
O3—H3A···S1	1.05 (2)	1.81 (3)	2.8163 (15)	159 (2)
